# Innovation in the realm of the unforeseen: a review of competence needed

**DOI:** 10.3389/fpsyg.2024.1166878

**Published:** 2024-03-20

**Authors:** Glenn-Egil Torgersen, Ole Boe, Leif Inge Magnussen, Dorothy Sutherland Olsen, Lisa Scordato

**Affiliations:** ^1^Faculty of Humanities, Sports and Educational Science, Department of Educational Science, University of South-Eastern Norway, Horten, Norway; ^2^Department of Industrial Economics, Strategy and Political Science, School of Business, University of South-Eastern Norway, Drammen, Norway; ^3^Nordic Institute for Studies in Innovation, Research and Education (NIFU), Oslo, Norway

**Keywords:** innovation, competence, the unforeseen, learning, educational design, systematic review, PRISMA

## Abstract

**Introduction:**

Our main research question in this article was: What are the competence structures for innovative processes? Both the nature of the unforeseen and innovation are related to something unknown, i.e., that competence needs to be developed to be able to handle situations and solutions that are not yet completely known. In our article, we address the question of how studies of innovation describe and use concepts of competence in various forms.

**Method:**

We performed a systematic review of the relation between the unforeseen and innovation. In this systematic review we followed the Non-Interventional, Reproducible, and Open (NIRO) Systematic Reviews protocol. The identification of studies via databases and registers was conducted in accordance with the Preferred Reporting Items for Systematic reviews and Meta-Analyzes (PRISMA) statement. The various types of competence found in the literature review were grouped in such a way that we could develop a structure to use as the basis for a new concept of competence needed in order to initiate and implement innovative processes. We refer to this overview of different competence types as designated competence structures for innovative processes. The searches resulted in the following number of abstracts, respectively Web of Science (2997), ERIC (454), and PsycInfo (550), meaning that a total of 3,768 abstracts were found to be relevant. The 3,768 abstracts were imported into the program Covidence for screening in the first search.

**Results:**

After completing the evaluation process in accordance with the PRISMA checklist and flow diagram, 32 abstracts were found to be relevant for our research question as they were related to competence for the unforeseen and innovation.

**Conclusion:**

Few of the studies we investigated specifically mention competence. Another finding is that the innovation literature describes the activities that should take place linked to what it refers to as competence. Thus, the innovation research field does not define what the competence is or what it consists of, at either individual or group level, but rather describes the activities that contribute to successful innovation in an organization with little emphasis on how this competence should be developed. Training perspectives are lacking when it comes to innovation literature perspectives on competence.

## 1 Introduction

Individuals, teams and organizations have a need to become better at dealing with what is not yet known, known as the unforeseen (Torgersen, 2015), or inventing something new that works better than what already exists, a process known as innovation (Van de Ven, 2017). Arabiun et al. (2024) postulate that the development of personal aspects should be taken into consideration when pursuing innovative processes. These aspects including looking to the future in life, personal independence in decision-making, etc. that can be the foundation for the development of entrepreneurial skills.

In the literature, innovation is most often researched as a *process* rather than the resulting new products and services. Studies of the innovation processes suggest that it is not linear, it is carried out by heterogeneous groups and consists, among other things, of a knowledge sharing and continuous learning (Lundvall and Johnson, 1994). Studies of innovation frequently mention the importance of learning and of knowledge, that organizations and individuals need to have. Often this knowledge is described as the results of research and development (R&D), of long experience, of collaboration with suppliers, competitors or the result of converting tacit knowledge to explicit knowledge (Nonaka and Takeuchi, 1995).

Rapid societal changes and unforeseen crises, such as pandemics, nuclear accidents, terrorist attacks and other existential threats, can have detrimental effects on social and economic systems and policy-making, and their long-term consequences remain largely uncertain (Saeverot, 2022). These changes can provoke situations (1) where one does not know what is going to happen, and (2) where something new must be devised that works better than what already exists. These two situations or phenomena are also referred to as “the unforeseen” (Torgersen, 2015) and “innovation” (Van de Ven, 2017). Handling both the unforeseen and innovation requires certain competences. The motivational psychologist White (1959) saw competence as the individual’s capacity to cope with demands from their environment. Utterback (1996) has looked more closely at the phenomenon of innovation and suggests that firms need to find the right balance of competence, they need strong core competence, but also a “a broad set of related and unrelated competences” (Utterback, 1996, p. 221).

Competence in handling uncertainty and unforeseen events has gradually become a clearer social and political task, both individually and in collaboration between different organizations (Tobias and Fletcher, 2000; Torgersen, 2018; Saeverot, 2022). Previous research shows that competence for flexibility will be important for handling both the unforeseen and innovation (Kanter, 1996, p. 117; Kaarstad and Torgersen, 2017). In acknowledging this need for competence, we must in turn accept the need for new learning in the workforce to provide new competence adapted to the new challenges. Learning and interaction during crises, with great risk and unpredictability, are examples of situations that require specific, adapted and different types of competences.

### 1.1 Converging on the unknown element of the unforeseen and innovation

Both the nature of the unforeseen and innovation are related to something unknown, therefore competence needs to be developed to be able to handle situations and solutions that are not yet completely known (Darsø, 2019). Darsø (2019) expresses this in her “innovation diamond.” This is a model that shows that an innovation process takes place between two extremes on a continuum scale, with the bases being knowledge and non-knowledge. “Knowledge” represents status or what we have competence and experience about today. “Not knowing,” in Darsø’s model, suggests a desirable end state, for example, new products or solutions, but that we do not yet know. Facilitation via interaction, communication, idea creation, terms and theories (concepts), under flexibility and positive and esthetic learning environments – to get from knowledge to non-knowledge – is central. Torgersen (2015, p. 330) combines Darsø’s (2019) knowledge–not knowledge scale with training models for both the unforeseen and the anticipated, where learning goals are more or less known. Such models can be aids for planning learning processes to develop competence to handle unforeseen events and carry out innovative processes. However, knowledge of which types of competence are desired to be developed is also needed.

In other words, there are several overarching similarities between the unforeseen and innovation. Therefore, we wanted to investigate which types of competence are highlighted as central to the development and implementation of innovative processes, and that may also be relevant for dealing with unforeseen events.

### 1.2 The research question

Our main research question was therefore: *What are the competence structures for innovative processes?* Our literature review investigates competence types that should be developed in order to initiate and implement innovative processes.

There is considerable learning potential in looking at how these two can be connected. Innovation is a process where unexpected things often happen (Van de Ven, 2017), but much of the classic innovation literature has focused instead on how systemic, structural, organizational etc. conditions affect such processes (Nelson and Winter, 1985). However, as found by Arabiun et al. (2024) entrepreneurial firms described by risk-taking, innovation and pioneering are more inclined to adapt their business and develop the essential capabilities to meet crucial requirements.

As our starting point for this identification process, we draw on the basic research on competence for the unforeseen conducted by Torgersen (2015, 2018). By querying the scientific literature, we aim to explore ways in which studies of the unforeseen and studies of innovation describe and use concepts of competence in various forms (Taleb, 2010; Weick and Sutcliffe, 2015).

### 1.3 The unforeseen and innovation – definitions

Seen in the light of pedagogy and organizational psychology, where identification of competence types and training for this is also emphasized, the unforeseen is often defined as: “A relatively unknown event or situation that occurs relatively unexpectedly and with relatively low probability or predictability to the individual, group or community that experience and handle the event” (Torgersen, 2015, p. 30; Torgersen, 2018, p. 27). A more in-depth definition, which also more specifically deals with the organizational perspective, is: “…a relatively unknown or rare event or situation primarily with negative or extreme consequences and a high degree of volatility, uncertainty, complexity, and ambiguity that occurs relatively surprisingly and unexpectedly, and the individual, group, organization or community that experience and manage the event failing to identify the event or giving it a relatively low or zero probability or predictability of occurring” (Herberg, 2022, p. 61). Unforeseen events have a nature that can be described as degrees of unforeseen along a continuum scale (Torgersen, 2015, p. 330). Common conditions that constitute degrees of the unforeseen are the degree of familiarity, identification of danger signals, and the time perspective in relation to the notification of the unforeseen events.

The term “Black Swans” (Taleb, 2010) is also often used as a metaphor for surprising and unexpected events (Johannessen, 2022). However, the unforeseen in our context is an overarching concept, covering underlying concepts with different shades of meaning, such as the “unpredictable,” “uncertain,” “unexpected,” “surprising,” “unknown,” “unimaginable,” “improbable” and “random” (Kvernbekk et al., 2015, p. 31).

Basic research studies reported in Torgersen (2015, 2018) provide some answers, where the core of the research is to identify which types of competence are needed.

Table 1 shows the main findings on competences for handling unforeseen events, in a preparedness perspective (Kaarstad and Torgersen, 2017). Torgersen (2018) elaborated on the concept of interaction (interaction under risk) in this context, and Herberg (2022) PhD thesis investigated and elaborated on these types of competence, several at unit level (in accordance with the NVQ system), and demonstrated, among other things, that ‘social support’ was one of the factors with the strongest weight.

**Table 1:** Competence types for handling unforeseen events.

Competence type	Short description
1.General preparedness	Coping and organization of basic skills
2.Emergency plans for the unforeseen	Existing plans for the management and prevention of particular unforeseen events
3.Understanding of “the unforeseen”	Employees’ and the organization’s definition, description and perception of particular unforeseen events that are relevant for the organization
4.Capabilities for handling the unforeseen	Coping and organization of basic skills particularly for unforeseen events
5.Improvisation	Employees’ and the organization’s ability to improvise and find creative solutions during unforeseen conditions
6.Flexibility	Employees’ and the organization’s ability and willingness to adapt their logistics and administrative system to the situation at hand
7.Identification of risk	The organization’s procedure to identify and pursue warning signs
8.Training and self-efficacy	Continuous competence development and training programs for unforeseen events. Focus on self-efficacy
9.Concurrent learning	The organization’s ability to emphasize observation and learning during events
10.Interaction	Employees’ and the organization’s ability to collaborate internally and externally when events occur
11.Social support	Support and communication between actors and from leaders to the actors during the incident
12.Emotions	Awareness of the importance of certain emotions for decisions and actions during unforeseen events

In addition, types of competence within emotional awareness were demonstrated as a new type of competence that is important when dealing with unforeseen events (Table 1).

### 1.4 Investigating how the scholarly literature describe and use the concepts the unforeseen and innovation

In our paper we address this question by systematically reviewing the scholarly literature on the unforeseen and innovation. The aim of the review is to increase our understanding of what distinct competences are necessary in order to initiate and engage in innovative processes. More generally, to be able to understand innovation processes and, not least, to develop learning programs to become better at initiating and implementing innovative processes, the appropriate types of competence must first be identified and specified. Pedagogical learning processes should entail both specific learning objectives, adequate training methods and forms of evaluation. Central to this is the use of didactic models that require learning objectives as a basis for educational processes. Therefore a thorough understanding and clarity about which types of competence are to be developed is fundamental (Tobias and Fletcher, 2000; Torgersen, 2015, 2018; Torgersen et al., 2020). Clearly identified competence types can thus be transformed into relevant learning objectives. Based on this, concrete learning methods, pedagogical facilitation, forms of evaluation of learning, and the enculturation process can be derived (Saeverot, 2022). The problem arises where it is difficult or impossible to determine in advance which competence is desired to be developed. Tommasi et al. (2021) found that current views of competences within the literature lack the practitioners’ perspectives, and underline the need to constantly monitor the ever-changing needs of industry and labor market transitions. Secondly, they established that education and training need to develop a culture that involves competence related to continuous learning and tools for detecting new learning needs.

One field of research we decided to include is innovation studies. Innovation studies do not say much directly about the unforeseen, but they do focus on novelty and disruption of the status quo. There is also an assumption that the past cannot be relied upon to provide all the answers for the future. Many innovation studies look at how organizations develop new products and services that do not yet exist and might provide interesting information on the kind of competence needed to do this, such as how to visualize the future and what to do if the future is different from the expected. Another feature of innovation research is that many studies have been carried out on innovation in practice and this might help us to move from the theoretical towards a more practical understanding of what is necessary and what is useful when working with the unforeseen.

The main contribution of this paper is therefore how it draws upon theoretical concepts from relatively unrelated fields of research – primarily educational science and innovation studies – to develop a better understanding of the competence and capabilities needed for people and organizations to address unforeseen problems and innovative processes.

### 1.5 The structure of the article

The paper is structured as follows: in section 2, we provide the conceptual background for the paper by first defining how the paper interprets the concept of competence, and we describe the state of the art with regard to research on the unforeseen, including relevant concepts from innovation studies. We outline the challenge of developing the necessary competence to deal with the unforeseen (Torgersen, 2015, 2018; Kaarstad and Torgersen, 2017; Torgersen et al., 2020, 2022; Herberg, 2022). We include a description of our method and the data resources used in this study. Thereafter we present a summary of our findings. The various types of competence found in the literature review are then grouped in such a way that we can develop a structure to use as the basis for a new concept of competence needed in order to initiate and implement innovative processes. The European Qualification (EQF) framework provides the translational basis for the Norwegian Qualification Framework (NKR). Carlsten et al. (2016) pinpoint that in translations the key qualifications of the unpredictable are left out of the Norwegian translation (i.e., at level six “to solve complex and unpredictable problems”). Therefore, in order to organize different competence types, we have chosen to use a traditional system, i.e., the National Vocational Qualifications (NVQ) Standards and Competence framework (Fletcher, 1994), which organizes competences in a hierarchical system, a system that is also utilized in working task analyzes and identification of competences (Annett and Stanton, 2000; Kirwan and Ainsworth, 2014; Armstrong, 2016). We refer to this overview of different competence types as designated competence structures for innovative processes.

## 2 The concept of competence in relation to innovation and the unforeseen

Competence belongs to everyday language and can therefore be described and understood in multiple ways. This is something that we also find traces of in the use of the term competence in the research literature. In this study, we adhere to the following definitions of competence in the areas of the unforeseen and innovation:

Competence is the combined knowledge, skills, abilities and attitudes that make it possible to perform relevant functions and tasks in line with defined requirements and goals (authors’ translation) (Lai, 2004, p. 48). Competence is considered a wide concept that embodies the ability to transfer skills and knowledge to new situations within the occupational area. It encompasses organization and planning of work, innovation and coping with non-routine activities. It includes those qualities and personal effectiveness that are required in the workplace to deal with co-workers, managers and customers (Fletcher, 1994).

Another definition of competence is: “…applied and applicable knowledge, skills and abilities that have use value in working life” (authors’ translation) (Nordhaug, 1993a, p. 19). White’s (1959) definition of competence as the individual’s capacity to cope with demands from their environment is perceived as generic and broad. In a working environment, competence can be seen as a result of knowledge, skills and ability (Nordhaug, 1993b, pp. 68–69) and is related to the different work tasks.

We find a different approach in Johannessen (2022), who suggests a systemic approach to continuous change in the innovation economy. This involves an overall view of competence, where the focus is not placed on the identification of specific types of competence. Instead, this approach emphasizes the adaptation of working methods, management and organization to conditions of uncertainty. This gives the organizations the possibility to cope with what is to come, and generate new ideas and products – even under unpredictable contexts.

According to Molander (1996), western scientific traditions have aimed at understanding the world through theory and there is a distinction between what people do and what has been seen as exceptions. Knowing in doing is related to attentivity to the world, the ever-moving bodily knowledge interrelating to the world, through reaction and reflection, fragments and holism, and between trust and critique. Knowing in action has a dialogic structure, the relationships between the embodied mind and the world. Knowledge then becomes a part of what is called competence. Competence (Dalin, 1993) is normally divided into technical and practical skills, practical or theoretical knowledge, and values that regulate our perception of the world and our choices. In addition come insights that stem from experience and work that can be difficult to articulate (Polanyi, 1966). The final piece of personal competence (Dalin, 1993) is related to networking and the use of other people’s competencies. Competence can be seen as manifest when it is showcased in practice situations, and latent as the capability to act when the world or situation changes. It furthermore involves the use of tools, interaction in heterogeneous groups and autonomous action.

In use, individual competence can show itself as action with or without deliberation (Løvlie, 2009) in each situation. Individual competence for the unforeseen (Herberg, 2022, p. 17) is connected to the concept of resilience:

*“…the intrinsic dynamic ability of individuals and collectives to prepare, absorb, recover, learn, adapt, and adjust functioning prior to, during, or following both short and long-term adversity, disruptive changes and disturbances, to sustain required operations in different domains and to provide acceptable functioning, positive outcomes and flexibility under unforeseen conditions.”* (Herberg, 2022, p. 17)

In a broader perspective, this competence can also be seen within the realm of structural optimism (Oeverman, 1999). The meaning of this is that resilience, the unforeseen and innovation intend to find new solutions and handle situations and processes in ways that have not been done before. In such a perspective, there is an underlying positive attitude associated with these concepts. The attitudes consist of being positive and actively going into difficult situations in order to solve them, instead of a more negative approach where the idea is that this cannot be solved.

Based on different designations for “competence,” there are also established systems for dividing levels of competence. These levels indicate an increasing degree of precision about which competence is needed or to be expressed. Such a level of precision is necessary in circumstances where the type of competence is to be used as a basis for education and training. Innovation literature has shown that the concept of competence is used at an overall level, and not as specific types. At the other end, individual character strengths are used as designations of certain characteristics or competences in handling the unforeseen. The question still remains as to whether there are any similarities or differences in the types of competence identified in the relationship between the unforeseen and innovation.

**Figure 1:**
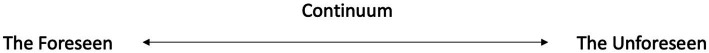
A continuum between the foreseen and the unforeseen.

**Figure 2:**
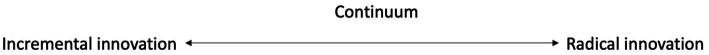
A continuum between incremental and radical innovation.

This study makes an attempt to clarify this through four “situations” connected through seeing how different degrees of the unforeseen can relate to different “degrees” of innovation, seen as a continuum between incremental and radical innovation (Figures 1, 2) (Torgersen, 2015; Darsø, 2019).

The unforeseen can be perceived as part of a continuum between the foreseen and the unforeseen, and creates different competencies needed by the agents/agencies involved.

We suggest that innovations also can be perceived as a continuum between incremental innovations and radical innovations. The different types of innovations affect the competencies needed by the agents/agencies involved.

### 2.1 Literature review

The challenge mentioned in the introduction motivated a systematic review of the academic literature. In this systematic review we followed the Non-Interventional, Reproducible, and Open (NIRO) Systematic Reviews protocol as advised by Topor et al. (2020).

The review process was inspired by the approach of Petticrew and Roberts (2006), who have outlined the rationale and methods of systematic reviews in the social sciences. They describe systematic literature reviews as “…a method of making sense of large bodies of information…” and “…of mapping out areas of uncertainty, and identifying where little or no relevant research has been done, but where new studies are needed” (Petticrew and Roberts, 2006, p. 2).

A review protocol was developed by the research team and pre-registered at the Open Source Framework (OSF) (Scordato et al., 2022). As stated by Munn et al. (2018), the Cochrane handbook (Higgins and Albrecht, 2022) refers to a systematic review as a review that “…uses explicit, systematic methods that are selected with a view to minimizing bias, thus providing more reliable findings from which conclusions can be drawn and decisions made” (Munn et al., 2018, p. 2). The identification of studies via databases was conducted in accordance with the Preferred Reporting Items for Systematic reviews and Meta-Analyzes (PRISMA) statement (Page et al., 2021).

### 2.2 Search strategy

The search and selection of articles was completed in four steps. First (step I), we carried out a keyword search in three databases: Web of Science Core Collection (1978–present), ERIC (Education Resources Information Center), and PsycInfo (Ovid). Development of the search strategy, queries, compilation and de-duplication of results were done by two specialist librarians working at the University of South-Eastern Norway (USN) University Library’s systematic search service. Controlled terms, synonyms and related terms for the concepts of “competence,” “the unforeseen” and “innovation”

were mapped by the librarians and reviewed by the authors. The search strategy was adapted to each database and their thesauri. Years for inclusion were set to 1978–present, and all languages were included. The search strategy was peer-reviewed according to the PRESS guidelines (McGowan et al., 2016) by a third librarian. The final literature search was conducted in June 2022. The literature search was based upon the following keywords illustrated in Table 2. The same keywords were also used for searching the ERIC and PsycInfo databases.

**Table 2:** An overview of the keywords used in the search strategy adapted to the syntax and the search history.

#	Searches	Results
1	(competen* or learn* or creativ* or improvis* or capab* or educating) (Topic)	2,435,302
2	(unforeseen or unexpect* or unpredict* or uncertain* or serendipity* or “black swan” or “cannot be foreseen” or “not foreseen” or “cannot be foreseen” or “cannot be expected” or “not expected” or “cannot be expected”) (Topic)	880,707
3	(innovation* or innovativeness or innovate*) (Topic)	267,433
4	#3 AND #2 AND #1 and Timespan: 1978–01-01 to 2022-12-31 (Publication date)	2,997

**Figure 3:**
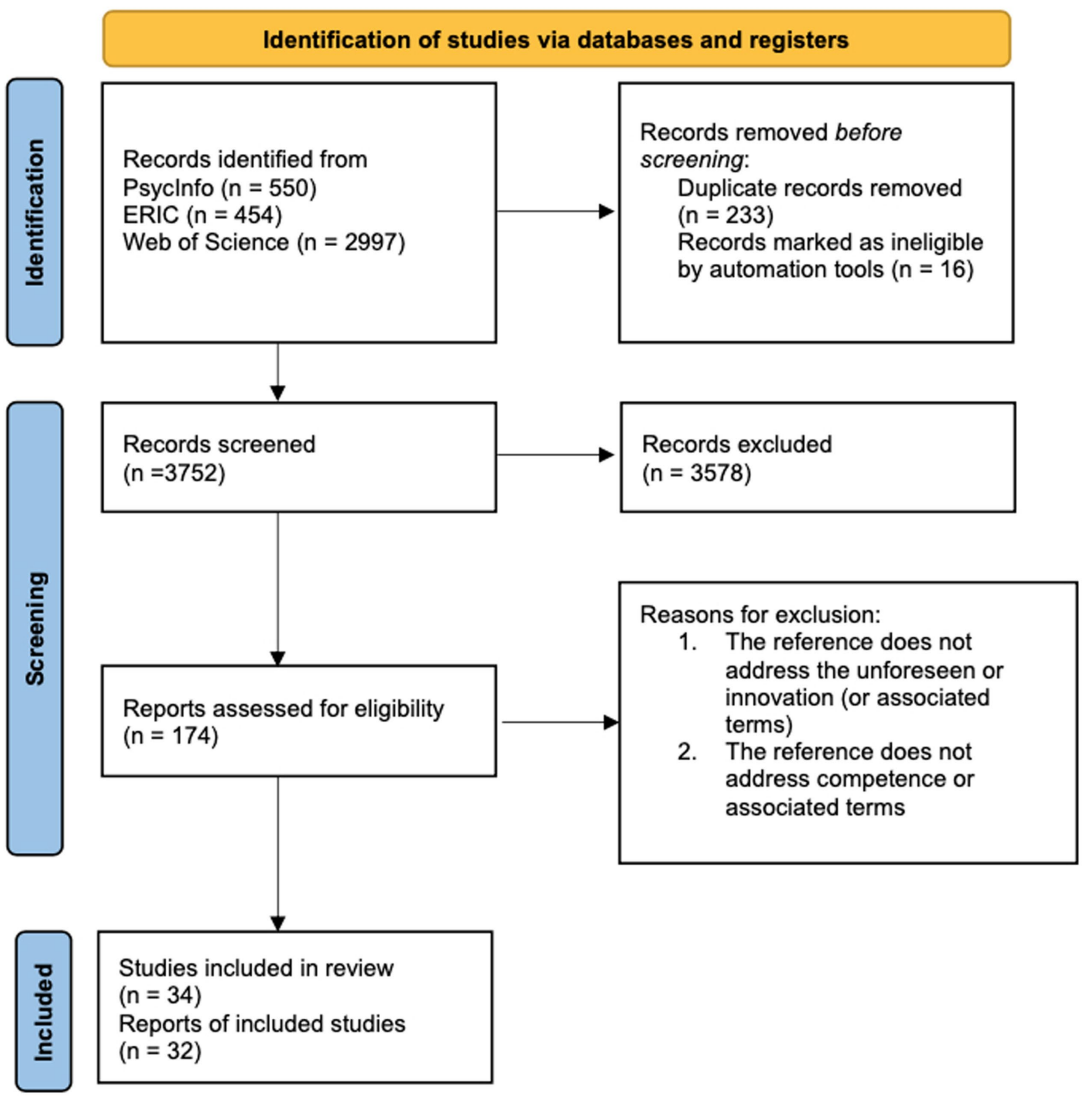
PRISMA flow chart.

The search yielded 4,001 references, of which 2,997 were retrieved from Web of Science, 454 from the ERIC database and 550 from PsycInfo. References were imported into EndNote, where duplicate references (*n* = 233) were removed. The 3,768 remaining references were imported into the reference screening and data extraction tool Covidence (Covidence, n.d.), where an additional 16 duplicates were identified and removed, resulting in a final number of 3,752 references from the first step. Figure 3 shows the PRISMA flow chart for our systematic review.

In a second step, we checked the level of inter-rater reliability (IRR) amongst the reviewers. Five reviewers screened the same first 200 titles and abstracts of the 3,752 identified references. The IRR was at approximately 70%, considering that the rating was done based on an ordinal scale: yes, maybe, or no, indicating a substantial agreement between the reviewers (Cohen, 1960; O’Connor and Joffe, 2020). Thereafter (step III), each reviewer screened an additional 750 abstracts, indicating if each reference was to be included, excluded or categorized as uncertain (“maybe”). The final decision to include or exclude uncertain references was taken by two of the reviewers. The two following reasons for exclusion were used: 1) the reference does not address the unforeseen or innovation (or associated terms); 2) the reference does not address competence or associated terms.

This step resulted in a total of 174 references to be further examined. These 174 abstracts were scrutinized to see whether they clearly covered the concept of competence seen in the light of the concepts related to the unforeseen and innovation. After screening the 174 abstracts that met the inclusion criteria, 34 were found to be relevant in relation to our research question as they specifically dealt with competence structures at a more fine-grained level and specifically dealt with the unforeseen and innovation. In a fourth and final step, the reviewers read the full texts of the 34 references, of which 32 were finally included in the analysis (Table 3). Two articles did not meet the criteria for inclusion and were therefore deleted from further review (see Table 4).

**Table 3:** Overview of the 32 articles related to competence for the unforeseen and innovation.

Competence type	Competence area/ profession	Author/s and year
1.Reflection, cognitive load, situational awareness	Management^a^/Military	Born and Lehner (2022)
2.(Capability), flexibility	Management	Bouhalleb and Smida (2020)
3.Learning adaptability, positive framing	Psychology^b^	Boulamatsi et al. (2021)
4.Creativity, divergent thinking, stretch-thinking loops, [plasticity]	Management/Public sector	Brooks and Curnin (2021)
5.Experience-based competence	Education and training^c^	Cederquist and Golüke (2016)
6.Leadership, creativity, self-efficacy, and risk-propensity	Education and training	Chell and Athayde (2011)
7.Improvisation	Management	Crossan (1996)
8.Accepting surprises	Management/Business	Cunha et al. (2013)
9.(Capability), serendipity	Organizational culture	de Rond (2014)
10.(Capability), flexibility (dynamic capabilities), interaction	Management/Business	De Toni et al. (2016)
11.Improvisation, embodied learning, creative thinking	Asthetic professions	Dou et al. (2021)
12.Dynamic capabilities, decision-making under uncertainty	Management/Business	Dyduch et al. (2021)
13.Creativity, understanding emotions	Psychology	Refaie (2013)
14.“Allgemeinbildung” (Enculturation), judgment and decision-making, care	Education and training	Elmose and Roth (2005)
15.Collective problem solving	Management/Business	Elsner (2018)
16.Collaborative relationship, collaborative leadership, communicating and sharing, information, trust formation, joint decision-making	Management/Business	Fanousse et al. (2021)
17.(Transversal skills) critical thinking, creative thinking, emotional, model thinking, prototyping	Education and training	Galeeva et al. (2018)
18.Cognitive load, problem-solving, innovative idea generation, avoiding, cognitive barriers	Management	Garbuio and Lin (2021)
19.Creativity, out-of-the-box thinking, prospective sensemaking	Management	Gattringer et al. (2021)
20.Flexibility, responsiveness, serendipity, creativity, willingness to change	Social sciences	Godfrey (2009) (PhD-thesis)
21.Dynamic safety capability, knowledge articulation, knowledge codification	Psychology	Griffin et al. (2016)
22.Capabilities, creativity, experiential learning	Education and training	Le Pontois and Jaillot (2021)
23.Creativity, understanding emotions, fear, recognition of creative ideas	Psychology	Lee et al. (2017)
24.Creativity, trialing, improvisation	Psychology	Leone (2020)
25.Knowledge articulation, tacit knowledge	Management	Nonaka (2007)
26.Capability, imagination, understanding of pedagogically rich scenario processes	Education and training	Rhisiart et al. (2015)
27.Situated (local) competence	Psychology	Scaratti and Ivaldi (2015)
28.Dynamic capabilities, entrepreneurial leadership (the ability to anticipate, challenge, interpret, decide, align, and learn)	Management	Schoemaker et al. (2018)
29.Dynamic capabilities, foresight (imagine possible futures)	Management	Semke and Tiberius (2020)
30.Disruptive creativity, unconventional innovative thinking and acting, theatrical skills	Education and training	Oparaocha and Daniil (2020)
31.Serendipity, metaphorical association	Psychology	Cunha et al. (2010)
32.Dynamic capabilities	Management	Pospichil et al. (2022)

a Management includes organizational dynamics, business, and crisis management in public. When using only management, this refers to management in general without any connection to a specific branch, organization, or area.

b Psychological processes related to competence development and learning.

c Education and training related to competence development and learning.

**Table 4:** Examples of articles not related to competence type for the unforeseen and innovation.

Overarching theme	Competence area/profession	Author/s and year
1.Learning process, new ways of collaboration, sensemaking, integration, collective learning	Pharmaceutical industry/Science	Dunne (2007) (PhD thesis)
2.Creativity, serendipity, insight	Management/Business	Moskowitz et al. (2006)

## 3 Typological findings and comments

Based upon reading the articles, we made resumés of how the competences were described and utilized in each article, as well as describing the context. Table 3 gives an overview of the competence type, competence area/profession, author/s and publication year. The competence types are reported exactly as they are written in the 32 different articles.

### 3.1 Identification of competence types for the unforeseen and innovation

In the overview of relevant abstracts (Table 3), we have included several terms that are easily identified as types of competence; however, there are many others that one might define as insight, understanding or an ability to work in a certain way. We have included these terms in Table 3 because they are all examples of things that can be learned, and that help organizations to find new ways of tackling unforeseen events. Some terms may require some explanation, such as entrepreneurial leadership, or entrepreneurial mindset. It is not necessary for an entrepreneurial manager to be an entrepreneur, but he or she must embody the typical traits of an entrepreneur, being willing to take risks and to be motivated by gains, financial or otherwise. In the context of the unforeseen, it is the willingness to take risks, perhaps only to see alternative ways of doing things, or to turn an organization upside down in order to make it better. This definition fits with Schumpeter (1942) and his claim that entrepreneurs are the actors who make change happen. Another term that perhaps needs some explanation is dynamic capabilities. This term became popular in studies of innovation after it was used by Teece et al. (1997) and is normally used to describe an ability that can be observed in some innovative organizations. It is the ability to continuously combine knowledge from within the organization with new knowledge from other sources outside the organization. The way Teece et al. (1997) describe it, it becomes a way of working, but is usually initiated by management.

Of the 32 articles, the competence descriptions in 15 of the articles are related to management, 7 are related to psychology, 7 are related to education and training, 1 is related to esthetic professions, 1 is related to organizational culture, and 1 is related to social sciences more generally.

Other terms in Table 3 give indications of where the knowledge might come from, such as from experience, experimentation, problem-solving and collaborative activities or local situated knowledge. Another group of terms in Table 3 indicate the importance of emotions and include the effects that feelings of fear might have on people trying to deal with the unforeseen. Other concepts draw upon skills from other fields such as the theater and the arts, and there is a strong focus on various ways of visualizing the future and developing the ability to improvise and find ways of getting used to surprises. There are also examples of terms which are related to planning and predicting and decision-making using various methods and new technologies. The term serendipity turns up in multiple abstracts and, although it may not be a clearly defined type of competence, several papers suggest that the abilities to recognize good ideas and to see the potential in an event are skills that can be developed. This list provides us with an interesting array of competences or abilities that might help with the unforeseen. Many of them confirm earlier studies of the unforeseen (Torgersen, 2015, 2018; Herberg, 2022), while others come from innovation studies, which, as we have mentioned earlier, are situations where the unknown and the unforeseen are an integral part of the process.

### 3.2 Examples of articles excluded due to superficial use of competence types

Below we give two examples of non-relevant articles found in the search strategy when screening the 34 abstracts (Table 4). The reason for their exclusion is that it was not specified in the two abstracts what competence is, for instance, whether it is creativity, improvisation, flexibility, and/or sensemaking.

Our interpretation is therefore that there was only a superficial description and use of the competence types used in these two articles. This was despite the fact that the abstracts related to the articles as a starting point suggested the opposite and were therefore accepted during the previous phases of the review process.

## 4 Discussion

Our aim with this article was to investigate the competence structures for innovative processes. However, few of the reviewed studies specifically mentioned competence. A possible reason for this could be that they use a slightly different ontology in describing competences. Another reason could be that they do not have a suitable professional background and that their scientific disciplinary background does not contain a terminology that can be related to competences.

Another finding is that the innovation literature describes the activities that should take place linked to what it refers to as competence. Thus, it is not often defined what the competence is or what it contains, at either individual or group level, but rather how the activities should appear in the organization. Therefore, very few studies within the literature field of innovation mention how this competence should be developed, i.e., there seems to be a lack of a training perspective when it comes to competences.

Von Tunzelmann (2009) discusses the differences between competencies and capabilities. The author relates this very much to the business aim. For a business, it is viewed as important to distinguish between static competence, which enables the firm to do what it does today, and dynamic capabilities, which enable the firm to adapt to changes in its environment and therefore survive in the future. von Tunzelmann (2009, p. 459) further differentiates between “capabilities (knowing-that, knowing-why) and procedural knowledge with competencies (knowing-how)”.

Many of the studies we investigated suggest that certain capabilities or capacities are necessary or that certain activities should be carried out. It appears that the idea of competence that might be developed in advance has not been considered. One of the reasons for this may be that the articles we have focused on are largely concerned with collective knowledge, shared culture and activities, with little mention of the individual. This focus on collective knowledge is common in studies of organizations and long-term change, while papers building on theories or traditions from psychology and educational science are more focused on individual competence.

Indeed, this lack of attention to individuals in studies of innovation has been registered by, among others, Felin and Foss (2005) and von Tunzelmann (2009). The former highlights the risk of assuming that the individuals who make up the collective are a homogeneous group and suggests that more research be done into the micro-foundations of change in organizations. It is no simple matter to build bridges from the collective level to micro-foundations, but some steps have been taken using a sociocultural perspective to understand collective activities (Engeström, 2014), which include individuals and larger groups, and the potential of using this in innovation studies has been suggested (Olsen, 2013).

We have nevertheless tried through our analysis to identify and to clarify which actual competence structures are relevant from the literature that we have investigated. We have done this by using theoretical methods within hierarchical competence structures (Fletcher, 1994).

However, Dou et al. (2021) state that research on creative thinking and innovative thinking has received scholarly attention, revolving around a specific type of thinking, but without providing a clear definition of creative thinking and innovative thinking. The creative and innovative thinking ability of choreographers positively correlates with the quality of dance creation. Improvisational dance has a creative connotation due to its randomness and uncertainty and it is therefore an essential practice for evaluating dancers’ creativity. The cultivation of creativity through improvisation and the creative and innovative thinking it embodies has been widely researched. The specific mode of thinking in dance creation is dominated by image thinking and supplemented by abstract thinking. Improvisational dance also follows a specific mode of thinking for dance.

Creative thinking can thus be seen as an original nature of thinking that emphasizes originality, divergence and appropriateness, and belongs to image thinking. Innovation thinking is a kind of regenerative thinking based on the application and popularization of new technologies and products, which emphasizes both social and economic benefits, and which aims to add value in terms of value addition; innovation thinking is part of abstract thinking.

### 4.1 Competence in innovation research: a foggy field?

As we have seen through our systematic review study, the research literature on innovation has not actually examined or reported which types of competence are highlighted as central to the initiating and implementation of innovative processes in a nuanced way.

The literature review of innovation and competences shows a lack of nuance in specifying the competences needed to bring about innovation. The researchers behind the articles and research projects content themselves with just hinting at overall types of competence, using words such as interaction, good management, corporate culture, creativity, future analysis and corresponding overall learning concepts. This may have something to do with their professional background, where such nuance is not part of the profession. A study by Fagerberg and Verspagen (2009) showed that approximately 58% of the respondents linked to a study on innovation had a financial background. The other respondents were distributed over a number of other subject areas that normally do not focus on specific types of competence or pedagogical systems and didactic methods to map or facilitate such competence for learning and training (such as engineers, historians, geographers, and sociologists, who made up just 5–9%). However, around 9% had a background in management and humanities, while less than 5% had a psychological background. Our systematic review suggests that these professions should therefore be included more within this innovation field, precisely to assist with research that can actually develop better competence within the unforeseen and innovation, to the benefit of both society and the profitability of companies.

Felin and Foss (2005) suggest that much of the management literature does not take into account the heterogeneity of the individuals who make up the workforce. They suggest that organizational routines and capabilities influence the way an organization will react to change, but say that we do not know enough about how these routines and capabilities arise or come into existence.

Felin and Foss (2005) also point out that different terminologies are used for individual-oriented characteristics, which contributes to ambiguity in thinking about, in particular, the concept of competence. As an example, the term micro-foundation is used instead of individual characteristics like “family.”

## 5 Conclusion

Our main research question in this article was: What are the competence structures for innovative processes? Through our literature review we have identified 32 different competence structures that all is important for innovative processes. However, these structures are not clearly defined in the literature and is not sufficiently exemplified and understood and used in training and learning processes. Knowledge about specific competence types is necessary to articulate leaning objectives and to facilitate learning processes with relevant teaching methods and for assessment of both process and outcomes. Hence, individuals and organizations can improve and become better at coping with initiating and implementing innovative processes.

We therefore consider the lack of clarity in how competence is treated by the innovation literature also to be an important finding. Related to this there is no clear way to transform innovation competence structures to the realms of the unforeseen.

### 5.1 Further research and limitations

A limitation in our systematic review is also that we did not investigate all possible databases, as we did not include the databases Academic, Scopus, Business Source Elite, and Social Science Premium Collection. Although the term *ambiguous* is found in the translation of the definition of the Norwegian definition of the unforeseen [det uforutsette] (Torgersen, 2015, p. 30; Torgersen, 2018, p. 27), the term was not put into the search string. Also, the fact that we did not read the full text articles that could be derived from the 3,768 abstracts might have interfered with or hampered our understanding of the phenomena we wanted to investigate. Furthermore, we are aware that there are other methodologies for categorizing competence into types, which could have resulted in other designations and nuances of the competence units. Another limitation is that our conception of industry and innovation is a Nordic one. This implies that our search string did not imply others relevant to innovation like Industry 4.0 (Lasi et al., 2014). However, the main content in the competence types for practical use in education and training would not change significantly if other methodologies had been used.

## Data availability statement

The raw data supporting the conclusions of this article will be made available by the authors, without undue reservation.

## Author contributions

G-ET, LM, OB, LS, and DO developed the theoretical basis and identified the keywords to be included in the search strategy. LS and OB developed the OSF protocol in collaboration with two specialist librarians at the University of South-Eastern Norway. All authors contributed to the article and approved the submitted version.
